# Intraflagellar transport trains and motors: Insights from structure

**DOI:** 10.1016/j.semcdb.2020.05.021

**Published:** 2020-11

**Authors:** Stephanie Webb, Aakash G. Mukhopadhyay, Anthony J. Roberts

**Affiliations:** Institute of Structural and Molecular Biology, Department of Biological Sciences, Birkbeck, University of London, Malet Street, London, United Kingdom

**Keywords:** Intraflagellar transport, Cilia, Microtubule, Kinesin, Dynein, Motor protein

## Abstract

Intraflagellar transport (IFT) sculpts the proteome of cilia and flagella; the antenna-like organelles found on the surface of virtually all human cell types. By delivering proteins to the growing ciliary tip, recycling turnover products, and selectively transporting signalling molecules, IFT has critical roles in cilia biogenesis, quality control, and signal transduction. IFT involves long polymeric arrays, termed IFT trains, which move to and from the ciliary tip under the power of the microtubule-based motor proteins kinesin-II and dynein-2. Recent top-down and bottom-up structural biology approaches are converging on the molecular architecture of the IFT train machinery. Here we review these studies, with a focus on how kinesin-II and dynein-2 assemble, attach to IFT trains, and undergo precise regulation to mediate bidirectional transport.

## 1 Introduction

**Fig. 1:**
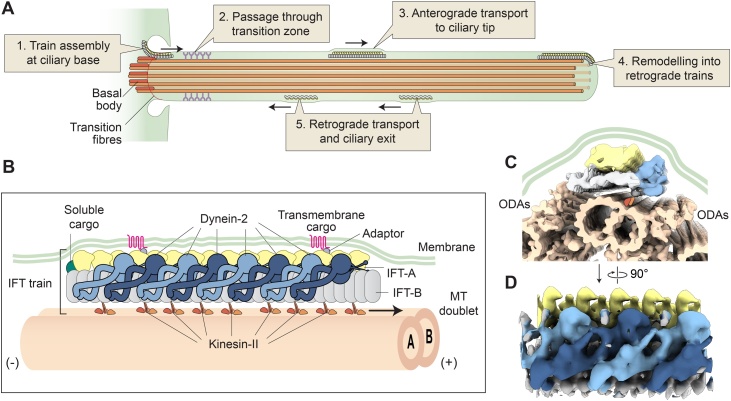
Overview of the IFT cycle and anterograde IFT trains. (**A)** Schematic of the IFT cycle. (**B**) Diagram of an anterograde IFT train moving to the tip the cilium. (+) and (-) indicate polarity of the microtubule (MT) doublet, which consists of a complete A-tubule attached to an incomplete B-tubule. Dynein-2 complexes along the train are shown in alternating dark and light blue for distinction. Length of the depicted train is 180 nm (anterograde trains vary in length from 100 to 700 nm in *C. reinhardtii* [28]). (**C**) Cross-section view depicting an anterograde IFT train moving in the confined space between the microtubule doublet, outer dynein arms (ODAs), and ciliary membrane. Created using EMD-4304 (sub-tomogram average of IFT-A), EMD-4303 (sub-tomogram average of IFT-B and dynein-2) [28], PDB-6SC2 (atomic model for dynein-2) [49], EMD-6872 (sub-tomogram average of microtubule-doublets and ODAs) [50], and PDB-4RH7 (used to model ODA stalks) [51]. Approximate position of kinesin is shown with orange triangles. (**D**) Sub-tomogram averages of IFT-A (yellow) at 33 Å resolution (EMD-4304) and IFT-B (grey) and dynein-2 (blue) at 37 Å resolution (EMD-4303) [28].

Cilia and flagella (here ‘cilia’) are multifunctional organelles that project from the surface of virtually every cell type in the human body and many other eukaryotic cells [1]. Their cylindrical structure is supported by nine microtubule doublets, which extend from the basal body at the plasma membrane (Fig. 1A). Motile cilia beat with a wave-like motion to generate fluid flow and propulsion. Their motility is essential for numerous biological processes, including the swimming of sperm and protozoa, generation of left-right body patterning, and mucus clearance in the respiratory tract [2,3]. Non-motile, primary cilia are recognised as the ‘signalling antennae’ of the cell [4,5]. They are rich in signaling molecules involved in functions as diverse as morphogenesis, appetite control, olfaction, and photoreception [[4], [5], [6], [7], [8], [9]].

To assemble and perform their critical functions, cilia use a conserved system to transport cargoes and signaling molecules to and from their tip [10]. This process of intraflagellar transport (IFT) involves multi-megadalton polymers, termed IFT ‘trains’, which move along the microtubule doublets [11,12] (Fig. 1A). IFT train movement is powered by the oppositely-directed motor proteins, kinesin-II and dynein-2 [[13], [14], [15]]. This review focuses on recent advances in understanding the molecular mechanisms of IFT trains and motors, which are of biomedical importance due to their dysfunction in a variety of human disorders (‘ciliopathies’) [16].

The size, complexity, and transient nature of the IFT machinery have made it difficult to study by structural biology techniques. However, recent studies have risen to these challenges using a variety of approaches and model organisms. For simplicity, in this review, we use human subunit names, except for the IFT proteins, where we use the well-established *Chlamydomonas reinhardtii* nomenclature (even when discussing orthologues in other species) [17,18]. Exciting progress has also been made in understanding the structures of the cargo-binding BBSome complex and the microtubule doublet, for which the reader is referred to recent articles [[19], [20], [21], [22], [23], [24], [25], [26]].

## 2 Overview of the IFT cycle

IFT trains (initially termed ‘rafts’) were first spotted between the ciliary membrane and microtubule doublets of *C. reinhardtii* cilia [11], and were confirmed as the vehicles of IFT by correlative light and electron microscopy [12]. Each train is a polymer of two large complexes, IFT-A and IFT-B (∼0.8 MDa and 1 MDa respectively in mammals), which interact with kinesin-II, dynein-2, and cargoes [17,27,28]. These interactions appear to be weak, as train components readily dissociate upon isolation from cilia [17,27,29,30]. *In vivo*, IFT trains assemble at the base of the cilium, near the transition fibres that connect the basal body to the membrane [17,[31], [32], [33]] (Fig. 1A, stage 1). IFT trains then pass through the ‘transition zone’; part of the diffusion barrier that separates the cilium from the cytosol [34] (stage 2). Anterograde train movement to the ciliary tip is powered by kinesin-II (stage 3) [12,35,36]. It occurs on the B-tubule of microtubule doublets [37], and can also occur on the singlet A-tubules found in the distal regions of primary cilia [[38], [39], [40]]. At the ciliary tip, anterograde trains remodel into retrograde trains (stage 4) [28,37,[41], [42], [43], [44]], which return to the ciliary base by moving along the A-tubule under the power of dynein-2 (stage 5) [37,[45], [46], [47]]. Here we focus on the molecular mechanisms underlying these events. Cargo binding to IFT trains is a highly regulated process, which has been reviewed recently [48].

## 3 Architecture of the anterograde IFT train

The structure of IFT trains has been studied by top-down approaches – illuminating train morphology [12,28,37,44,54,55] – and bottom-up approaches – providing insights into the molecular components of the train (reviewed in [18]). To date, the most detailed insights into the architecture of IFT trains *in situ* have come from sub-tomogram averaging, providing views of the *C. reinhardtii* anterograde train at 33–37 Å resolution [28]. The overall positions of IFT-A, IFT-B, and dynein-2 have been mapped [28], exploiting the availability of *C. reinhardtii* mutants [56,57].

**Fig. 2:**
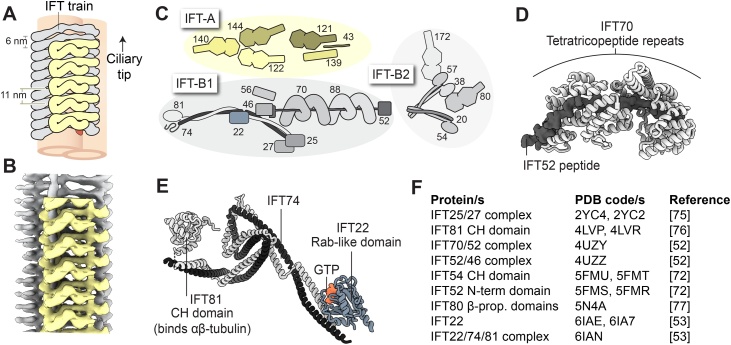
The IFT-A and IFT-B complexes. (**A**) Schematic of an anterograde IFT train, viewed from the ciliary membrane. Dynein-2 is omitted for clarity. IFT-B repeats every 6 nm along the long axis of the train, whereas IFT-A repeats every 11 nm [28]. (**B**) Sub-tomogram averages of IFT-A (EMD-4304) and IFT-B (EMD-4303) [28]. Dynein-2 density from EMD-4303 (shown in Fig.1D) has been removed to provide a clearer view of IFT-B. (**C**) IFT subunits, which form the IFT-A, IFT-B1, and IFT-B2 sub-complexes. β-propeller domains are depicted as heptagons. (**D**) Crystal structure of the IFT70/52 complex from *C. reinhardtii* (PDB-4UZY) [52]. (**E**) Crystal structure of the IFT22/74/81 complex from *Trypanosoma brucei* (PDB-6IAN) [53]. CH; calponin homology. (**F**) Table of IFT-B crystal structures.

Cryo-electron tomography shows that the IFT-A and -B complexes display distinct periodicities within the polymer: IFT-B repeats every 6 nm, whereas IFT-A repeats every 11 nm [28] (Fig. 2A,B). The IFT-B polymer tends to extend by several repeats beyond IFT-A, and does not require stoichiometric IFT-A in order to form [28]. These data illuminate IFT-B as the backbone of the train [28], consistent with genetic evidence that IFT-B is critical for IFT and cilium formation [17,58,59].

The IFT-A complex lies directly underneath the ciliary membrane [28], compatible with its role in the ciliary import of a variety of membrane proteins [60,61] (Fig. 1B). As discussed below, IFT-A is also important for retrograde IFT [[62], [63], [64], [65], [66], [67]]. The IFT-A complex consists of six proteins (Fig. 2C). Three of these (IFT144/140/122) form a core, important for the stability of IFT-A, whereas the others (IFT139/121/43) form a peripheral complex [60,66,68]. While no molecular resolution structural information is yet available for IFT-A, four of its proteins (IFT144/140/122/121) have a domain organization reminiscent of membrane coat proteins (two N-terminal β-propeller domains, followed by C-terminal tetratricopeptide repeats) [[69], [70], [71]].

IFT-B lies between IFT-A and the microtubule track [28] (Fig. 1B). It comprises at least 16 different proteins, which biochemical and proteomic studies have classified into two sub-complexes: IFT-B1 (10 subunits) and IFT-B2 (6 subunits) (Fig. 2C) [[72], [73], [74]]. Crystal structures of a number of important IFT-B proteins have been solved in the last 10 years (Fig. 2D-F) [52,53,72,[75], [76], [77]].

These structures, together with biochemical studies, have revealed two organizational principles within the IFT-B1 sub-complex. First, two proteins (IFT81/74) form a twisted heterodimeric coiled coil [53] that scaffolds several domains involved in cargo binding, including the calponin-homology domain of IFT81 (mediating tubulin binding [76,78]); the Rab-like domains of IFT22 and IFT27 and jelly-roll fold of IFT25 (involved in the BBSome pathway [79,80]); and the heterodimeric domains of IFT46 and IFT56 (implicated in Kif17 and axonemal dynein binding [[81], [82], [83]]) [53] (Fig. 2C,E). Second, IFT52 bridges multiple partners. Its central region interacts with the tetratricopeptide repeat proteins IFT70 and IFT88, while its C-terminal domain binds IFT46, and its N-terminal GIFT domain associates with IFT-B2 (Fig. 2C,D) [52,72].

Less is known about the structure of the IFT-B2 sub-complex, but a pair of coiled coil proteins (IFT57/38) also plays a central role in its formation (Fig. 2C). Their coiled coil interacts with IFT54/20, while their N-terminal calponin-homology domains bind to IFT172 and IFT80 [72,73]. IFT172 and IFT80 both consist of two N-terminal β-propeller domains, followed by C-terminal tetratricopeptide repeats. The IFT80 β-propellers have been crystallised, and, interestingly, tend to homodimerize *via* the C-terminal α-helical domain [77]. Atomic structures of the IFT-A, -B1 and -B2 complexes are highly anticipated to shed further light on how IFT trains polymerize and generate binding sites for cargoes, adaptors, and motors.

## 4 Navigating the microtubule doublet and transition zone

At ∼50 nm across [28], the anterograde IFT train is similar in width to the microtubule doublet and scarcely fits between the outer dynein arms that power the beating of motile cilia (Fig. 1C). Together with the closely apposed ciliary membrane, the shape of the anterograde IFT train is likely to position kinesin-II on the protofilaments of the B-tubule near the junction with the A-tubule [22,28]. The width of the IFT train also has implications for how it crosses the transition zone, which is characterized by Y-shaped links between the microtubule doublets and ciliary membrane. As the Y-links appear to emerge near the junction between the A- and B-tubules [84], they may clash with the position of the anterograde IFT train seen in the body of the cilium [28]. It will be interesting to decipher if passage through the transition zone requires a conformational change in the IFT train or Y-link. Recent studies show that mutations in IFT-A and dynein-2 subunits can perturb the localization of transition zone proteins, highlighting a connection between IFT and transition zone integrity [[85], [86], [87]].

## 5 The anterograde motor

**Fig. 3:**
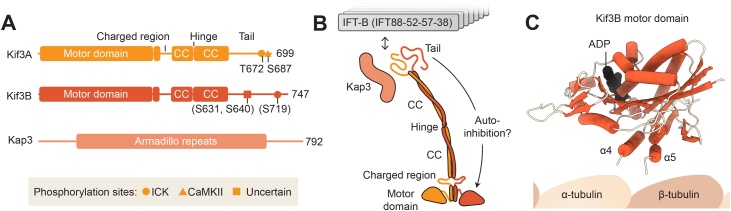
Heterotrimeric kinesin-II, the anterograde motor. (**A**) Domain organization of human heterotrimeric kinesin-II, Kif3. Major phosphorylation sites in the human subunits are shown (putative sites in parenthesis) [[108], [109], [110], [111], [112]]. Kif3A numbering is given relative to canonical 699 amino acid isoform. A 702 amino acid isoform also exists containing 3 additional amino acids in the charged region. In the literature some phosphorylation sites are given using this numbering (making the ICK site T675 and the CaMKII site S690). In Kif3B, the residue equivalent to S631 in the *C. reinhardtii* ortholog is phosphorylated by CrCDPK1 [112]. (**B**) Schematic of Kif3 and its interaction with IFT-B [113]. (**C**) Crystal structure of the Kif3B motor domain bound to ADP (PDB-3B6U) .

The principal motor driving anterograde movement of IFT trains is heterotrimeric kinesin-II (Kif3 in mammals) [12,35,36,88,89]. Within the heterotrimer, the Kif3A and Kif3B subunits each comprise an N-terminal motor domain, coiled-coil segments mediating heterodimerization, and putatively disordered tail (Fig. 3A**,**B). The third protein, Kap3, contains Armadillo repeats. Kap3 was found to be dispensable for Kif3 motility *in vitro* [90] but required for proper IFT *in vivo*, suggesting it plays a regulatory role [91].

Kif3 composition is further complicated in mammals, in which another chain, Kif3C, can pair with Kif3A in place of Kif3B [92,93]. Only Kif3AB is demonstrated to function in IFT with Kap3, whereas Kif3AC has cytoplasmic functions in neurons [92,93]. This suggests that Kif3B contains elements important for incorporation of the kinesin into the IFT machinery. While Kif3AB also has cytoplasmic roles, for example in mRNA and vesicle transport [94,95], its subunits appear to have co-evolved with cilia and IFT is likely its ancestral function [13].

The Kif3B motor domain, revealed by an unpublished crystal structure from the Structural Genomics Consortium (PDB-3B6U), has a classic kinesin organization (Fig. 3C). It consists of a β-sheet flanked by α-helices and contains the sites of ATP hydrolysis and microtubule binding. Based on high sequence identity (∼64 %), Kif3A’s motor domain is likely to share a highly similar structure. Nonetheless, the Kif3A and Kif3B motor domains display interesting differences in their motile properties, the extent of which varies among species [[96], [97], [98], [99]].

Sequence analysis suggests that Kif3A and Kif3B are held together *via* three segments of coiled-coil: one proximal to the motor domains [100], and two longer segments separated by a di-glycine hinge (Fig. 3A,B). Folding about this hinge is likely to give rise to the extended and compact forms of Kif3 identified by sedimentation analysis [101]. Assuming the standard 0.15 nm per residue, the total length of coiled coil in Kif3 is approximately 29 nm, compatible with observations from rotary shadow electron microscopy [90,101]. A region of two heptad repeats in the distal coiled coil is important for nucleating heterodimerization [102,103]. The distal coiled coil may be stabilized by the binding of Kap3 [104], whose Armadillo repeats have been predicted to wrap around the coiled coil [105].

C-terminal to the coiled coil is the putatively disordered tail, which is longer in Kif3B (∼160 residues) than in Kif3A (∼110 residues). The tail may be a focal point for regulation. It is widely held that Kif3 is inhibited at the ciliary tip, enabling it to be recycled back to the cell body. Akin to other kinesins [106], Kif3 is thought to be auto-inhibited by folding of its tails or coiled coil onto the motor domains [97]. In *C. reinhardtii*, binding of Kap3 has been found to relieve auto-inhibition [98]. In mammals, the Kif3 heterotrimer appears to be more tightly auto-inhibited as additional factors are required for its activation in the case of mRNA transport [107]. The Kif3 tail contains multiple phosphorylation sites (Fig. 3B). For example, the Kif3A tail is reported to be phosphorylated by the kinases ICK, PKA, and CaMKII [[108], [109], [110], [111]], whereas the *C. reinhardtii* Kif3B ortholog (FLA8) is phosphorylated by CrCDPK1 (a homolog of CaMKII) [112].

How does Kif3 attach to the IFT train? Insight into the binding site of Kif3 on the train has been obtained using a visible immunoprecipitation (VIP) assay [113]. This revealed that Kif3 interacts with a four-protein complex (IFT88/52/57/38) that represents the interface between IFT-B1 and IFT-B2. The tail of Kif3B is important for this interaction [113]. In *C. reinhardtii*, phosphorylation of Kif3B by CrCDPK1 disrupts the interaction with IFT-B, showing how kinesin-train association can be regulated [112]. In the sub-tomogram average of the anterograde IFT train, density connecting to the microtubule doublet is observed, probably corresponding to Kif3 [28]. It is interesting to note that the surface of IFT-B is closely apposed to the microtubule in anterograde trains [28], suggesting that the ∼29 nm of coiled coil in Kif3 is either folded into the train or lays across multiple repeats. This may contribute to Kif3 motility regulation upon binding to the IFT train; a process that awaits elucidation.

### 5.1 Homodimeric kinesin-II

While heterotrimeric kinesin-II is the sole motor for anterograde IFT in most organisms, in *Caenorhabditis elegans* chemosensory cilia, a striking division of labour occurs [114,115]. Here, heterotrimeric kinesin-II (Klp20-Klp11-Kap1) propels IFT trains through the transition zone and along proximal segment of the cilium, but is gradually replaced by a homodimeric kinesin-II (OSM-3) which drives faster movement along the distal segment [116]. The molecular mechanism underlying train handover from heterotrimeric kinesin-2 to OSM-3 is still emerging. Recent data implicate two kinases (DYF-5 and DYF-18, homologs of MAK and ICK) in the transition [117,118].

The study of *C. elegans* OSM-3 has also proved fertile ground for understanding kinesin-II regulation. OSM-3′s two identical subunits consist of an N-terminal motor domain, coiled-coil segments mediating homodimerization, and a tail. Akin to Kif3, a hinge exists within the coiled coil, and sedimentation analysis suggests that OSM-3 can exist in compact or extended forms [119]. The compact form is an auto-inhibited state, in which the tail is thought to interact with the motor domains [119,120]. Reconstitution studies revealed that binding of four IFT-B1 proteins (IFT88/70/52/46) is sufficient to activate OSM-3 *in vitro* [121]. Notably, while IFT70 alone is sufficient to relieve OSM-3 auto-inhibition, the other three IFT-B proteins are required for maximal motor velocity [121]. These data provide a paradigm for IFT motor regulation by the IFT train [121].

In vertebrates, a homodimeric kinesin-II (Kif17) also localizes to cilia, but does not function in conventional IFT [88,122]. Kif17 has been found to interact with a different site of the IFT train compared to *C. elegans* OSM-3 [81], and appears to be carried into cilia as a cargo; a process that is regulated by its nuclear localization signal [123]. *In vitro* studies show that Kif17 exhibits auto-inhibition [124] and, when activated, is a processive motor [124,125], but further studies are required to elucidate its function in cilia. For example, it is possible that Kif17 is involved in the direct transport of membrane proteins [126].

## 6 The retrograde motor

**Fig. 4:**
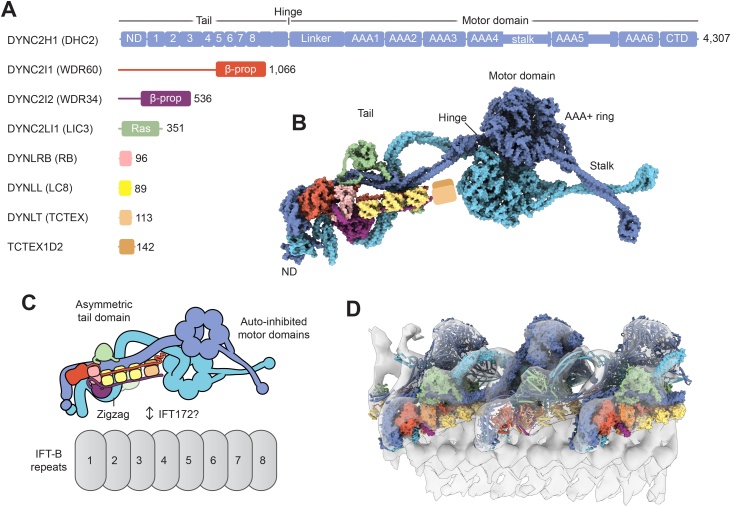
Dynein-2, the retrograde motor. (**A)** Domain organization of the human dynein-2 subunits. The heavy chain (DYNC2H1) is divided into a tail and motor domain. The tail consists of a N-terminal domain (ND) and a series of α-helical bundles [49]. The motor region contains a lever-like linker domain, six AAA+ modules (AAA1−6) and a C-terminal domain (CTD). AAA1 is the ATPase site that drives dynein-2 movement, whereas AAA2 and AAA3−4 are ATP and ADP binding sites respectively [51]. The microtubule-binding domain lies at the tip of a coiled-coil stalk. The canonical isoform of human DYNC2H1 is 4307 amino acids; a non-canonical isoform featuring a 7 amino acid insertion in AAA5 also exists. Trypanosomatids feature two distinct dynein-2 heavy chains that form a heterodimer [129]. (**B**) Cryo-EM structure of the human dynein-2 complex [49]. Subunits are shown in surface representation, except for the flexibly attached subunits DYNLT and TCTEX1D2. (**C**) Diagram showing how the asymmetric tail domain and auto-inhibited motor domains of dynein-2 spread out over ∼8 IFT-B repeats. (**D**) Docking of the dynein-2 cryo-EM structure (PDB-6SC2) [49] into the sub-tomogram average of IFT-B and dynein-2 (EMD-4303; transparent isosurface) [28]. Individual dynein-2 complexes are shown in alternating surface and cylinder representation for distinction. Adapted from [49].

The conserved motor for retrograde IFT is dynein-2, a large protein complex consisting of at least 8 different proteins in mammals (Fig. 4A) (reviewed in [14,15,127]). Dynein-2 activity is tightly regulated, as it is carried as a cargo on anterograde trains before being activated for retrograde transport at the ciliary tip. Recent advances in understanding the structural mechanism of dynein-2 include the crystal structure of the dynein-2 motor domain [51], a structural mechanism for dynein-2 auto-inhibition [128], visualization of inhibited dynein-2 on anterograde IFT trains [28], and a cryo-EM structure of the dynein-2 complex at 3.9–4.5 Å resolution [49].

The cryo-EM structure of the human dynein-2 complex shows how it is built around two copies of a >4000 amino acid heavy chain (DYNC2H1) [49] (Fig. 4B). The N-terminal region forms the tail, which is involved in dimerization and binding of associated subunits. The C-terminal region forms the ring-shaped AAA+ motor domain [51], whose functional elements are described in Fig. 4A**.**

The two motor domains of dynein-2 intrinsically tend to stack against each other in a conformation that inhibits their ATPase activity and motility [128]. This auto-inhibited “cross-legged” state of dynein-2 [128] is conserved in cytoplasmic dynein-1 [130], suggesting it is an ancient form of dynein motility regulation. Auto-inhibition of dynein-2 was proposed to facilitate the transport of dynein-2 to the ciliary tip by kinesin-II [128]. Supporting this model, *in vitro* assemblies of dynein-2 and kinesin-II were found to move efficiently along microtubules in the kinesin direction [128]. Sub-tomogram averaging provided *in vivo* evidence for inhibited dynein-2 on anterograde IFT trains and showed how its stalks point away from the microtubule [28]. These data shed light on how dynein-2 is transported to the tip of the cilium in an inactive state.

The two copies of the dynein-2 heavy chain are highly asymmetric in the tail [49] (Fig. 4B,C). The tail asymmetry is generated by an unusual stoichiometry sub-complex of intermediate and light chains [49]. The C-terminal β-propeller domains of the intermediate chains (WDR34 and WDR60) each bind a copy of the heavy chain, and are heterodimerized by their N-proximal regions and an array of light chains (one DYNLRB dimer, three DYNLL dimers, and a presumptive DYNLT-TCTEX1D2 heterodimer) [29,49,[131], [132], [133], [134], [135], [136]]. This sub-complex of intermediate and light chains stabilizes straightening of one heavy chain in the tail and steers the other into a zigzag conformation, which tailors dynein-2′s structure to the repeat of the IFT-B polymer [49].

The specific IFT-B proteins that interact with dynein-2 on anterograde trains are still emerging. Molecular genetic studies implicate the C-terminal region of IFT172 in anterograde transport of dynein-2 or its activation at the ciliary tip [44,137,138]. Interestingly, dynein-2 heavy chain immunoprecipitated with IFT172 migrates differently by SDS-PAGE compared to that from crude extract, suggesting that it could be differentially modified [44]. Additional IFT-B proteins have been associated with dynein-2 in trypanosomes (IFT22/25/27) [[139], [140], [141]] and mammalian cells (IFT25/54/57/74/88/172) [86].

Cryo-electron tomography shows that dynein-2 repeats at 18 nm intervals along the anterograde train (*e.g.* every 3 IFT-B repeats), and successive complexes pack against each other [28]. Docking the high-resolution cryo-EM structure of dynein-2 [49] into the sub-tomogram average [28] reveals that the light-intermediate chain (DYNC2LI1) of one complex contacts the motor domain of its neighbour (Fig. 4D). These data suggest that dynein-2 complexes may load cooperatively onto the assembling IFT train at the ciliary base, as binding of one dynein-2 complex to the train would create extra binding surface for the next.

## 7 IFT train remodeling and turnaround

In *C. reinhardtii*, an average anterograde IFT train is approximately 300 nm long, and comprises 25 IFT-A repeats, 50 IFT-B repeats, and 13 dynein complexes (in addition to a less well-defined number of kinesin-II complexes) [28]. Thus, the theoretical mass of an anterograde IFT train is >80 MDa. This remarkable value is comparable to one of the largest molecular machines in the cell, the nuclear pore complex (66–125 MDa, depending on species).

**Fig. 5:**
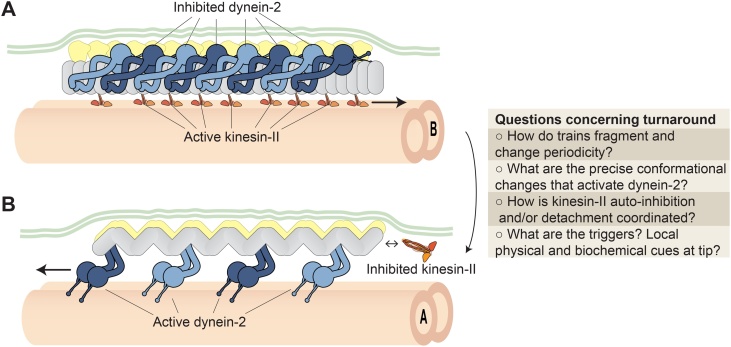
IFT train turnaround. Open questions surrounding the conversion of anterograde trains (**A**) into retrograde trains (**B**) at the ciliary tip. Within the retrograde train, the relative positions of IFT-A and IFT-B and the structure of dynein-2 are unknown.

In contrast to the nuclear pore complex, the architecture of IFT trains is linear and extremely dynamic. Upon reaching the ciliary tip, anterograde trains “remodel” into retrograde trains, involving multiple events whose mechanism is still emerging [28,37,[41], [42], [43], [44]]. Fluorescence microscopy suggests that anterograde IFT trains at least partially fragment at the tip [41,42,142], consistent with observations of more trains leaving the tip than entering it (for example [41,43,[142], [143], [144]]). Although retrograde trains have not proved sufficiently ordered to allow sub-tomogram averaging to date, raw tomograms suggest they have a distinctive periodicity, displaying a loose zigzag or helicoid shape (pitch ∼40 nm) (Fig. 5B), in contrast to the densely packed anterograde train (Fig. 5A) [28,37]. The remodelling event – which occurs within seconds [41,42] – also coordinates inactivation of kinesin-II with activation of dynein-2, and can promote the release of cargo [48].

What might be the biochemical and biophysical mechanisms underlying these events? The large-scale remodelling between anterograde and retrograde trains at the tip implies the need for an energy source or the release of internally stored strain within the IFT train polymer. The former mechanism could involve tip-localized biochemical modification of the train or motor subunits. For example, the IFT train involves a number of GTPases (IFT22, IFT27, RabL2) [145], whose nucleotide status could affect train assembly or cargo binding [146]. Kinases have been reported to localise at the tip, including ICK in mammals and CrCDPK1 in *C. reinhardtii*, both of which target kinesin-II [110,112]. Furthermore, ubiquitination has recently been found to regulate coupling of cargo to the IFT machinery [147,148].

In *C. reinhardtii*, kinesin-II has been found to detach from the train at the tip of the cilium and return to the base predominantly by diffusion, whereas in metazoa, kinesin-II appears to be principally transported on retrograde trains (discussed in [41]). It has been reported that dynein-2 can fully detach from the train at the tip [28], at least transiently [41,42]. Release of kinesin-II or dynein-2 from the train could destabilize the train polymer, since dynein-2 makes multi-valent interactions across multiple IFT-B repeats [49,28] (Fig. 4C,D). Indeed, the anterograde IFT train might be inherently unstable and require apposition between the ciliary membrane and microtubule doublet to remain intact. In this scenario, running off the end of the microtubule at the ciliary tip could be sufficient to evoke anterograde train fragmentation and remodeling. The involvement of local biochemistry and the physical environment of the ciliary tip in train remodeling is not mutually exclusive, and both may contribute to the turnaround event.

The conformation of the IFT train polymer is likely to be coupled with the activity of kinesin-II and dynein-2. For instance, the distinct periodicity of the retrograde train would destroy the multi-valent binding site for inhibited dynein-2, facilitating dynein-2′s reconfiguration into an active conformation [49,28,128]. The relative positions of IFT-A and IFT-B in the retrograde train are not yet clear. A wealth of data demonstrates that IFT-A is required for retrograde transport [[62], [63], [64], [65], [66], [67]], whereas it is partially dispensable for the formation of anterograde trains [28]. In the simplest model, IFT-A may bind to and activate dynein-2 directly. Alternatively, IFT-A could be an essential structural component of the retrograde train and thus indirectly required for dynein-2 activation. In either case, dynein-2 must undergo a major conformational change to become active, involving unstacking of its motor domains [128,28] and changes in its asymmetric tail domain [49]. Major open questions surrounding the mechanism of IFT train remodeling are summarized in Fig. 5.

## 8 Conclusion

Twenty-seven years since the discovery of IFT [11], top-down and bottom-up studies are converging on the functional architecture of the IFT train and motors. In the coming years, we can anticipate a more complete fusion of these approaches, which should provide atomic models for IFT train assembly, locomotion, and turnaround, and assist in the precise manipulation of IFT within living cells. We can also look forward to a more complete understanding of regulated cargo binding by IFT trains, and its interplay with the geometries of the ciliary base, transition zone, and tip. The integrative methods developed to address these challenges will likely help in understanding other transient, mesoscopic machines in the cell.
